# A Rare Case of Rapunzel Syndrome Presenting with Perforation Peritonitis

**DOI:** 10.7759/cureus.42440

**Published:** 2023-07-25

**Authors:** Sarthak Uttam, Shesh Kumar, Shivali Singh

**Affiliations:** 1 General Surgery, Uttar Pradesh University of Medical Sciences, Etawah, IND

**Keywords:** gastric perforation, trichophagia, trichotillomania, gastric trichobezoar, rapunzel syndrome

## Abstract

Rapunzel syndrome with gastric perforation is an extremely rare presentation of trichobezoars of the stomach. Trichobezoars may vary greatly in presentation ranging from benign symptoms like vague abdominal pain and anorexia to grave complications like perforation peritonitis. A sincere evaluation of any underlying psychiatric illnesses, usually trichotillomania and trichophagia, holds the key to preventing recurrences in patients of trichobezoar. A 15-year-old adolescent female presented with signs and symptoms of enteric perforation with a history of trichotillomania and trichophagia. Exploratory laparotomy of the patient revealed anterior gastric perforation with a huge gastric trichobezoar that extended into the duodenum and jejunum, hence establishing the diagnosis of Rapunzel syndrome.

## Introduction

Trichotillomania is defined as an irresistible urge to pull out hair from various parts of one’s body like scalp, eyebrows, and genital regions. It is part of a spectrum of disorders called obsessive-compulsive disorders (OCD) and is thought to be largely related to disorders of anxiety. It most commonly manifests in young adolescent females. There have been studies that have demonstrated genetic anomalies associated with trichotillomania [1].

Trichobezoar is an organized collection of hair and intestinal secretions in the alimentary canal. It most commonly forms in the stomach but can appear in other parts of the gastrointestinal tract as well [2]. Usually, trichobezoars are associated with trichotillomania and trichophagia (morbid habit to chew hair). Although rarely trichophagia can lead to trichobezoar without the presence of trichotillomania [3].

Rapunzel syndrome is a rare entity in which a trichobezoar presents with a long tail of hair that extends into the duodenum, jejunum, ileum, or even colon. The overall incidence of gastric trichobezoars has been estimated to be around 0.4-1% in the general population. Cases of trichobezoar have been reported in literature very infrequently, and Rapunzel syndrome is extremely rare and less than 50 cases have been reported in medical literature to date [4]. This syndrome is named after the girl with long tresses in the fairy tale written by the Grimm Brothers in 1812 [5]. The Rapunzel syndrome was first reported in two cases by Vaughan et al. in 1968 [6].

This is a case report of a 15-year-old female with Rapunzel syndrome presenting with gastric perforation.

## Case presentation

A 15-year-old female presented to the emergency department with complaints of sudden onset pain in the abdomen five days back and abdominal distension for the past 15 days, which was gradual in onset and progressive in nature, associated with non-projectile vomiting which was watery. The patient had not been passing flatus or stool for the past three days. She had been experiencing intermittent, indolent pain in the upper abdomen and a gradual decrease in appetite for the past six months. Her mother gave the history of her habit of pulling and chewing her hair since she was five years old, which the patient also confirmed.

**Figure 1:**
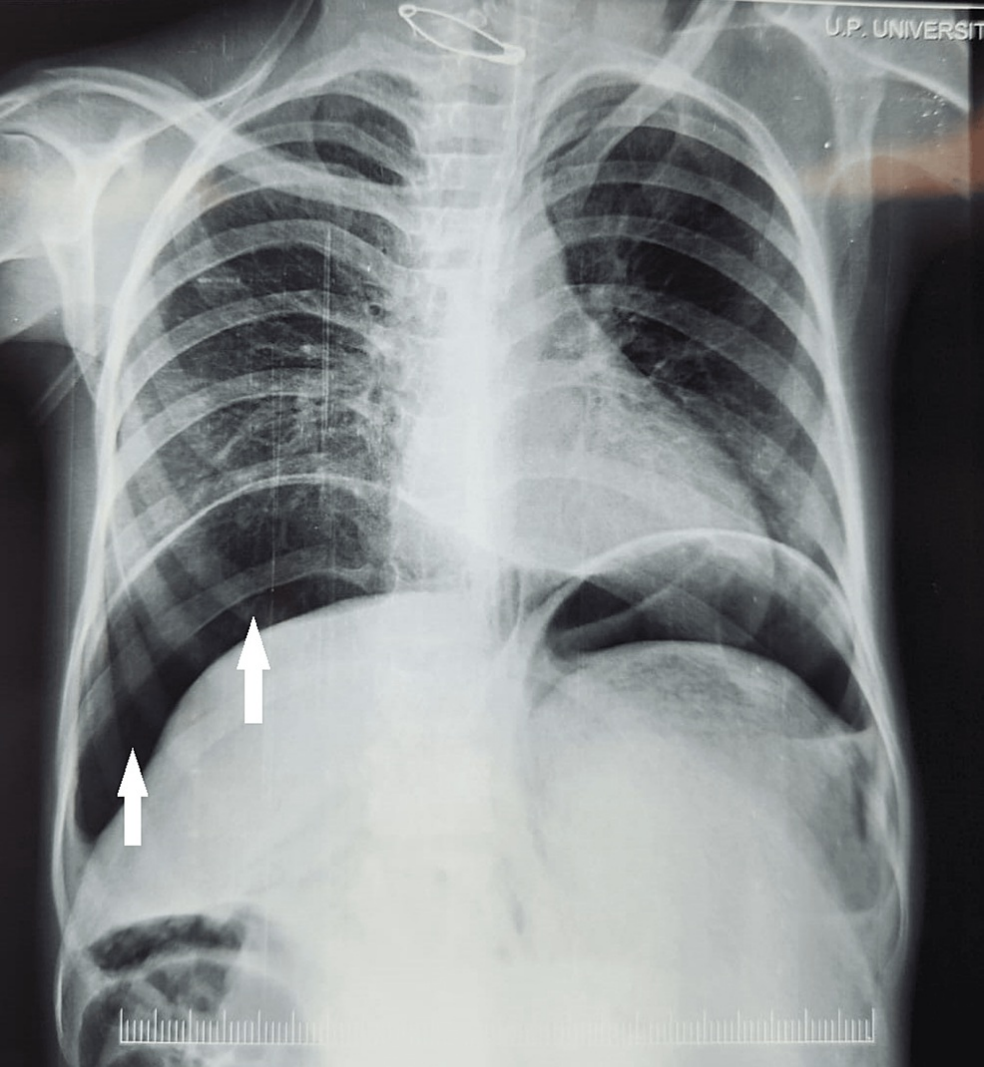
Gas under diaphragm seen in erect chest X-ray (marked by white arrows).

In the general examination, the patient was found to be anemic and distressed. On abdominal examination, generalized distension and guarding were elicited suggestive of enteral perforation. An immediate X-ray of the chest and abdomen was obtained in which gas under the diaphragm (Figure 1) was seen and a diagnosis of enteral perforation peritonitis with impending septic shock was established.

**Figure 2:**
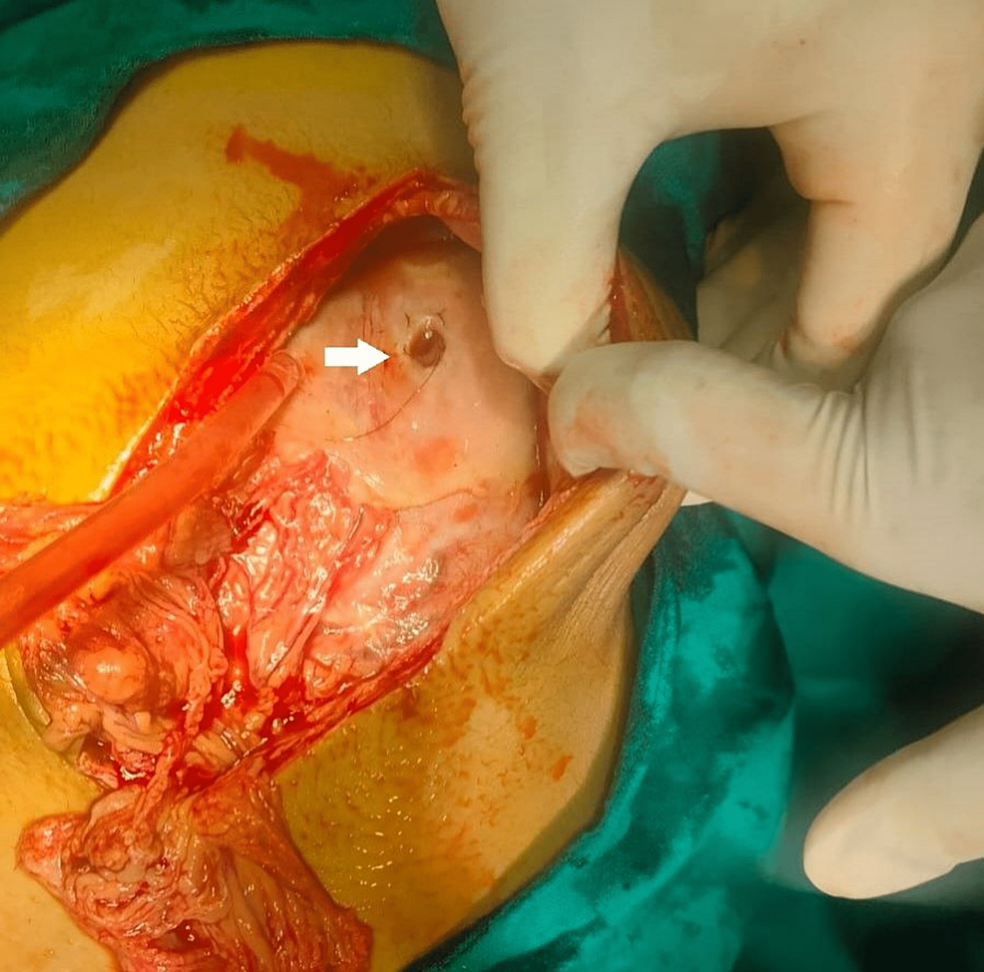
Laparotomy revealed a 1.5 × 1.5 cm perforation of anterior gastric wall with a strand of hair lying over it (pointed by white arrow).

**Figure 3:**
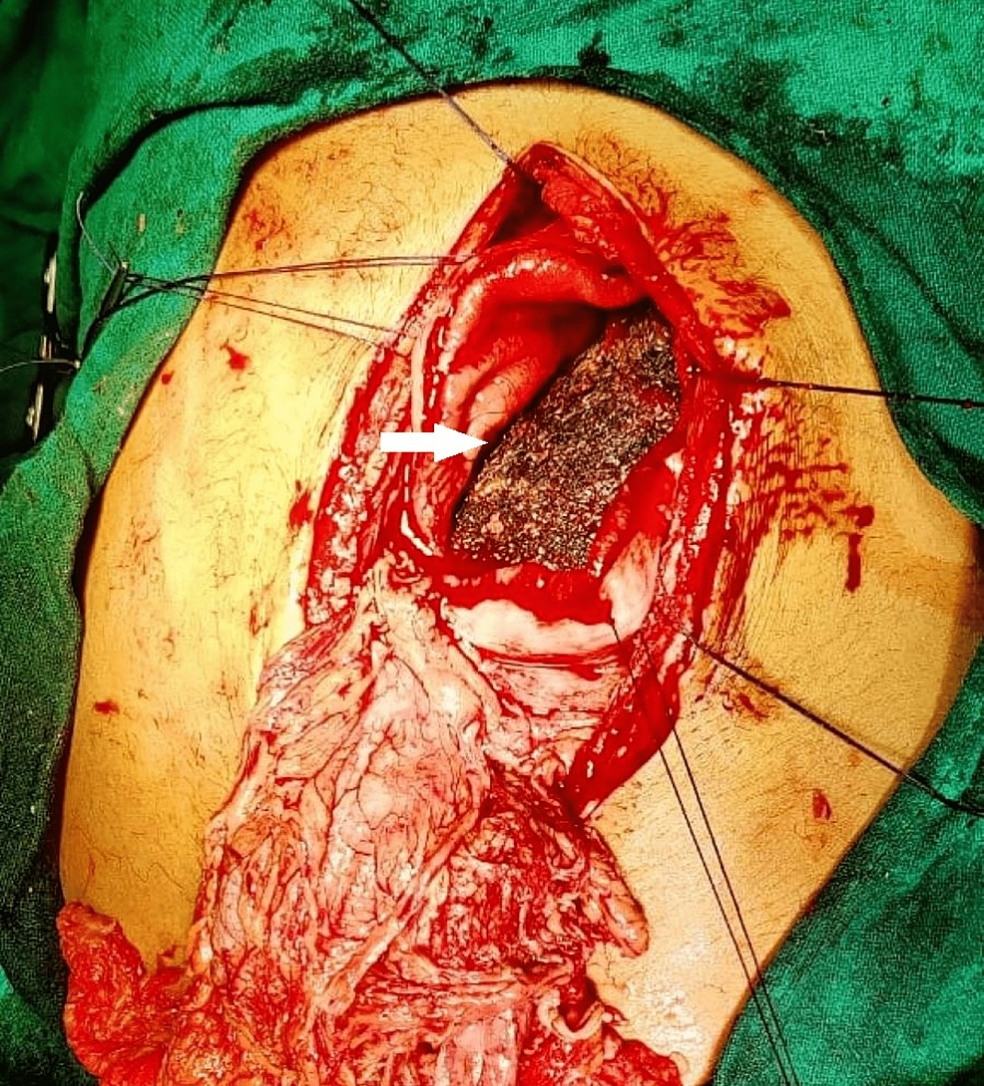
Anterior gastrotomy revealed a massive lump of hair in the entire gastric lumen.

**Figure 4:**
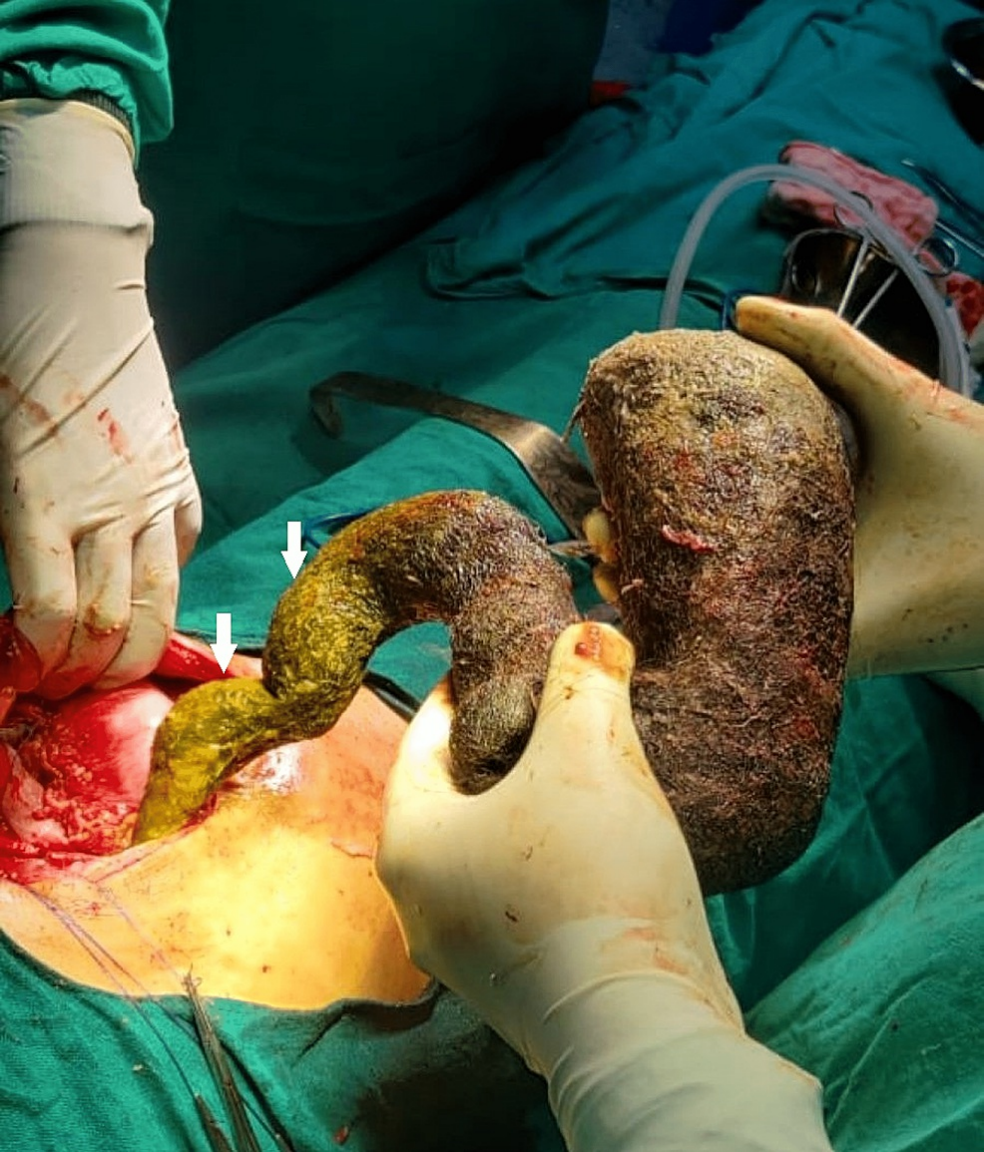
The trichobezoar extended into the duodenum and jejunum as marked by the bile staining evident in the distal end of the specimen.

**Figure 5:**
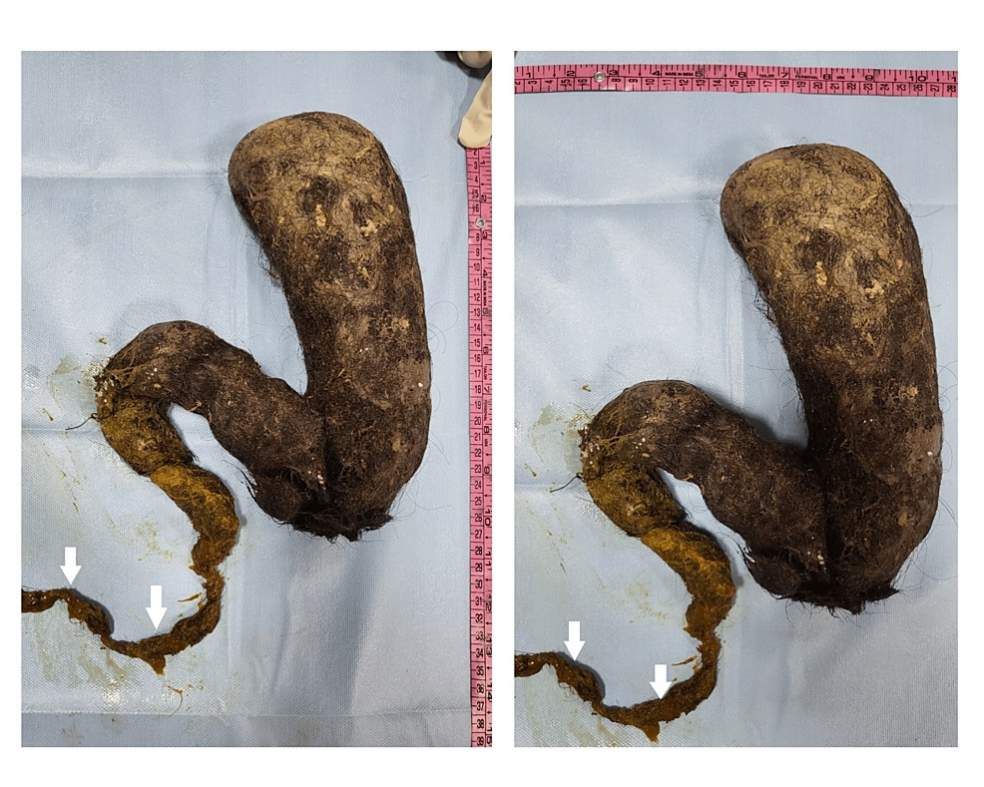
Trichobezoar specimen after removal. Notice the extension of the tail as seen in Rapunzel syndrome.

The patient was immediately shifted to the operating room with ongoing resuscitation for exploration of the abdominal cavity. Intraoperative findings revealed a 1.5 × 1.5 cm anterior gastric perforation with a strand of hair lying over it (Figure 2). Anterior gastrotomy revealed a huge trichobezoar (Figure 3) extending into the duodenum and jejunum. Its maximum width was 8 cm and an intragastric length of 27 cm, but it extended further into the duodenum and jejunum for another 30 cm (Figure 4 and Figure 5). An en-bloc removal of the cyst was successful after some difficulty and extension of the gastrotomy wound (Video 1). Gastric mucosa appeared normal and gastrotomy was closed in two layers after excising ulcer margins for H. Pylori status and histopathology. An omental patch was fixed over the gastrotomy suture line and after a thorough peritoneal lavage, drains were placed and a fascial closure of the abdomen was done leaving the skin incision open for a delayed primary closure. The patient was kept in the surgical ICU for two days where she recovered well and was started with oral liquids on postoperative day 2. She further showed good recovery, and on day 5, skin closure of the wound was done. She was discharged on day 7. On follow-up, the patient was evaluated by a psychiatrist, and her counseling sessions with a psychologist were planned.

**Video 1:** Removal of huge gastric trichobezoar in a patient with trichotillomania and trichophagia (Rapunzel syndrome).

## Discussion

The word "bezoar" has been derived from the Arabic word "badzehr" meaning antidote in the context of usage of animal substances and stones to counter poison. These practices were largely abandoned in the nineteenth century but the term "bezoar" carried on and denotes any foreign indigestible substance that accumulates in the gastrointestinal tract [7].

Bezoars can be composed of plant derivatives (phytobezoars), medications (pharmacobezoars), milk (lactobezoars), or hair (trichobezoars). Of these, phytobezoars are by far the most common variant. Several factors predispose to the development of bezoars like impaired motility of gastric musculature (gastroparesis), psychiatric illnesses like pica, trichotillomania, trichophagia, and long-term intake of certain medications like anticholinergics and opiates [7]. 

Trichobezoars usually present clinically with symptoms of pain in the abdomen, vomiting, decreased appetite and weight loss, or an abdominal lump. Complications from trichobezoars include gastrointestinal obstruction, bleeding, perforation, or nutritional deficiencies. Gastric perforation is a rare presentation of trichobezoars [8].

Diagnosis of such patients is dependent on clinical history and examination supported by radiological and endoscopic evaluation. Although CT scans and sonography have high rates of accuracy in diagnosing such patients, this case was taken to the operating room as soon as evidence of enteric perforation was established on the abdominal X-ray skiagram. Endoscopy has recently gained popularity as a therapeutic tool in upper gastrointestinal bezoars. A retrospective study conducted by Gökbulut et al. and Mihai et al. found 86.5% and 88.7% success rates, respectively, in treating bezoars endoscopically [9,10].

A multi-disciplinary approach to such cases including a surgeon, gastroenterologist, dietician, and psychiatrist is the right way forward.

## Conclusions

Rapunzel syndrome with gastric perforation should be managed in lines of any other viscus perforation with special attention to the patient’s psychiatric evaluation in order to avoid recurrences. During the early course of their disease such patients usually present with subtle upper gastrointestinal symptoms, and a careful history taking and attention to any peculiar psychosocial habits, especially in an adolescent female, should urge the physician to consider gastrointestinal bezoars among other probable pathologies.
